# The effect of turmeric (Curcumin) supplementation on cytokine and inflammatory marker responses following 2 hours of endurance cycling

**DOI:** 10.1186/s12970-014-0066-3

**Published:** 2015-01-21

**Authors:** Joseph N Sciberras, Stuart DR Galloway, Anthony Fenech, Godfrey Grech, Claude Farrugia, Deborah Duca, Janet Mifsud

**Affiliations:** Sport Nutrition graduate from the University of Stirling, 74, San Anton Court, Pope John XXIII street, Birkirkara, BKR1033 Malta; Health and Exercise Sciences Research Group, School of Sport, University of Stirling, Stirling, Scotland; Department of Clinical Pharmacology and Therapeutics, University of Malta, Msida, Malta; Department of Chemistry, University of Malta, Msida, Malta; Department of Pathology, University of Malta, Msida, Malta

**Keywords:** Immunity, Interleukins, Natural polyphenols

## Abstract

**Background:**

Endurance exercise induces IL-6 production from myocytes that is thought to impair intracellular defence mechanisms. Curcumin inhibits NF-κB and activator protein 1, responsible for cytokine transcription, in cell lines. The aim of this study was to investigate the effect of curcumin supplementation on the cytokine and stress responses following 2 h of cycling.

**Methods:**

Eleven male recreational athletes (35.5 ± 5.7 years; W_max_ 275 ± 6 W; 87.2 ± 10.3 kg) consuming a low carbohydrate diet of 2.3 ± 0.2 g/kg/day underwent three double blind trials with curcumin supplementation, placebo supplementation, and no supplementation (control) to observe the response of serum interleukins (IL-6, IL1-RA, IL-10), cortisol, c-reactive protein (CRP), and subjective assessment of training stress. Exercise was set at 95% lactate threshold (54 ± 7% W_max_) to ensure that all athletes completed the trial protocol.

**Results:**

The trial protocol elicted a rise in IL-6 and IL1-RA, but not IL-10. The supplementation regimen failed to produce statistically significant results when compared to placebo and control. IL-6 serum concentrations one hour following exercise were (Median (IQR): 2.0 (1.8-3.6) Curcumin; 4.8 (2.1-7.3) Placebo; 3.5 (1.9-7.7) Control). Differences between supplementation and placebo failed to reach statistical significance (p = 0.18) with the median test. Repeated measures ANOVA time-trial interaction was at p = 0.06 between curcumin supplementation and placebo. A positive correlation (p = 0.02) between absolute exercise intensity and 1 h post-exercise for IL-6 concentration was observed. Participants reported “better than usual” scores in the subjective assessment of psychological stress when supplementing with curcumin, indicating that they felt less stressed during training days (p = 0.04) compared to placebo even though there was no difference in RPE during any of the training days or trials.

**Conclusion:**

The limitations of the current regimen and trial involved a number of factors including sample size, mode of exercise, intensity of exercise, and dose of curcumin. Nevertheless these results provide insight for future studies with larger samples, and multiple curcumin dosages to investigate if different curcumin regimens can lead to statistically different interleukin levels when compared to a control and placebo*.*

## Background

Research supports a role for nutritional interventions to maintain immune function in the post-exercise period [1-5] It is also widely recognized that endurance exercise stimulates an increase in circulating cytokines in the post-exercise period [6,7]. These cytokines include interleukin 1 beta (IL-1β), interleukin 6 (IL-6), interleukin 8 (IL-8), interleukin 10 (IL-10), and interleukin 1 receptor antagonist (IL1-RA). These cytokine responses following exercise do not mainly originate from circulating monocytes, but may influence secretion of other cytokines from cells which form part of the immune system [8,9]. The post-exercise rise in IL-6 is unrelated to muscle damage, but serves as a messenger from myocytes to increase hepatic glycogenolysis [10-12]. Interestingly, the release of IL-6 in the post-exercise period appears to be dependent upon carbohydrate availability [12].

IL-6 is a cell messenger which affects many cells and systems, such as lymphocytes, leads to the release of the anti-inflammatory hormone cortisol, and stimulates release of acute phase proteins and glucose from the liver [13] IL1-RA and IL-10 transcription are mediated by high IL-6 concentrations [14]. These immunomodulatory mechanisms result in a decreased amount of circulating Type 1 T-helper (Th1) cells [15]. This suggests that regular high volume exercise shifts the CD4 positive T lymphocyte profile from Th1 towards Th2. Th1 cells help neutralize intracellular infective agents like viruses and bacteria which are responsible for upper respiratory tract infections (URTI). Specific interleukins, involved in cellular immunity, are also inhibited by the increase in IL-6 [16]. Inhibition of IL-1 is mediated through IL1-RA, and IL-6 appears to blunt the effect of TNF-α, while the effects of interleukin 12 are countered by IL-10 [17]. Thus, factors that can modify the post-exercise cytokine response could assist in maintenance of immune function in athletes.

Cytokine transcription is mediated by the transcription factors NF-κB and activator protein 1 (AP-1) [18]. Activation of NF-κB is induced by several immunity mediators, cell signaling intermediates, and reactive oxygen species [19]. Curcumin found in the rhizome Curcuma longa (turmeric), is an anti-oxidant and anti-inflammatory, long used as a traditional herbal medicine [20-22]. It attenuates the activation of NF-κB and IκB kinase in cancer cell lines [23]. Researchers observed that curcumin inhibits the activity of IκB kinase and decreases the activity of NF-κB in intestinal epithelial cells [24]. Shisodia et al., reported that the activity of curcumin also affects the AP-1 pathway, and Akt signaling [25]. In a study on rats, curcumin was shown to reduce IL-6, IL-1β, and TNFα levels following eccentric exercise [26]. These authors concluded that curcumin may promote recovery following repeated strenuous activity. Curcumin has also been shown to affect numerous physiological pathways, including inflammation, and play important roles in pathological conditions, including diabetes and arthritis, as reviewed elsewhere [27-29].

These observations with curcumin in cell and animal models, leads us to hypothesize that curcumin supplementation in humans could reduce cytokine release following exercise. An acute blunting of the cytokine response to exercise may provide a strong basis for longer term studies examining a role for curcumin on immune function and recovery during periods of strenuous exercise training. The current study, therefore, aimed to observe the effects of curcumin supplementation on interleukin and other inflammatory marker responses following two hours of cycling in a low glycogen state.

## Methods

Eleven recreationally active males (regular weekly aerobic activity during the last year for at least 3 h, mean age 35.5 ± 5.7 years; mean W_max_ 275 ± 56 W; mean weight 87.2 ± 10.3 kg; mean height 1.78 ± 0.07 m) volunteered to participate in the study. All of the participants gave their written informed consent to participate in the study which was approved by the ethics committees of the University of Stirling and University of Malta. Athletes were recruited from those attending talks held at sport clubs in Malta. Participants were screened for suitability prior to the experimental trials, including a medical visit by a licensed general practitioner. None reported a history of auto-immune disorders or medical conditions which could affect the results. Moreover they were not on medication or high dose vitamin C and/or vitamin E intake. Participants reported being free from infection for at least 4 weeks prior to the trial, and were in a steady period of endurance training. The number of participants needed was calculated by sample size testing based on literature review. Power was set at 80%, p < 0.05, with a difference in population means of 2 pg for interleukin 6, and standard deviation of 2 pg. This gave an approximate sample size of 8–10. The sample sizes and results obtained in studies listed in the review of Fischer, 2006 [30], on interleukins and exercise were also taken as a guide.

Participants were taught how to use diabetic nutritional exchanges to comply with the pre-trial prescribed diet. Preliminary measurement of lactate threshold and maximum workload were obtained together using a Computrainer lab ergometer (Racermate, Seattle, USA) and Lactate Scout (EKF-Diagnostics, Magdeburg, Germany). The Lactate Scout was validated prior to each test using the standard solution provided by the manufacturer. Lactate was measured by skin pricking every three minutes on the computrainer® lab; following which power was increased by 30 W. This continued until volitional fatigue or until the athlete was unable to maintain a cadence of 70 rpm. This was defined as the maximum workload. Subjects were then allocated either to the curcumin supplement or placebo in a double blind randomized cross-over fashion. Subjects performed three trials in total (supplement/placebo and control) in which they exercised for 2 h at a power output equivalent to 95% of their lactate threshold, to ensure completion of the trial task. Supplement or placebo was taken for three days prior to the trial day, and finally on arrival at the clinic for the trial. Following a one week wash-out period the trial was repeated with supplementation/placebo accordingly. An identical further trial served as a control and was held following a further week without any supplementation. The control arm of the study was scheduled after the two experimental trials in an effort to minimize data loss from curcumin and placebo trials, through athlete drop out. In addition, participants undertook a supervised one hour interval training session on a cycle ergometer in the afternoon, two days prior to each trial, in an attempt to lower muscle glycogen stores. Participants were then assigned a diet containing 2.3 ± 0.2 g/kg carbohydrate, 1.0 ± 0.2 g/kg fat, and 1.3 ± 0.2 g/kg protein. This diet was aimed at minimising carbohydrate replenishment following training two days prior to the trial. Participants returned to their habitual diet immediately after the trial. Participants were requested to refrain from strenuous physical activity for 24 h prior to trials.

**Figure 1:**
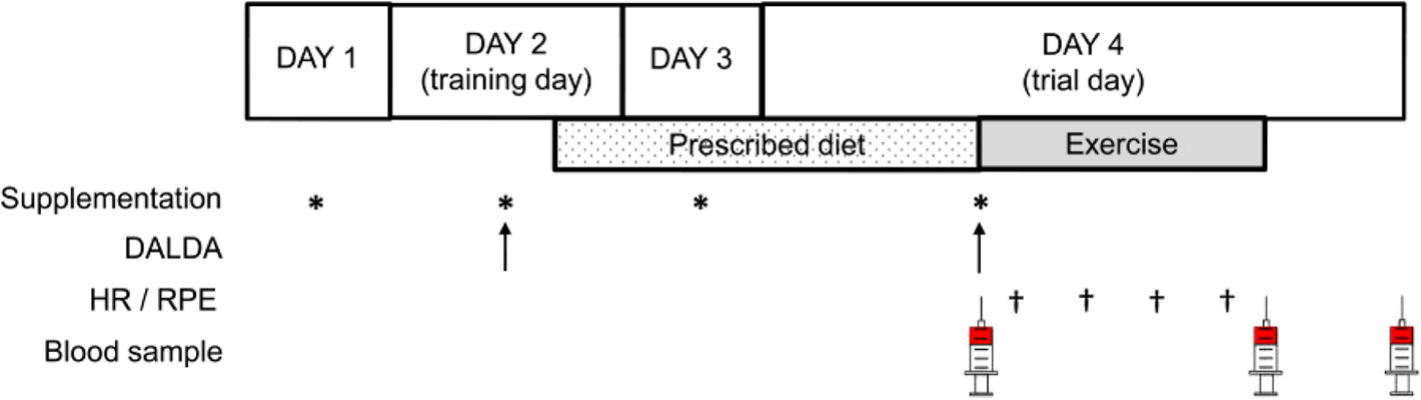
**Trial flow chart detailing sequence of events, supplementation days, and blood sampling.** DALDA – daily analysis of life demands in athletes, HR – heart rate, RPE – rating of perceived exertion.

Upon arrival at the laboratory for the trials a cannula was inserted in an antecubital vein. Blood samples (20 ml, 4 serum and 2 EDTA tubes) were taken just before the exercise trial, immediately after completing the two hours cycling, and one hour following the cessation of exercise (Figure 1). A pedaling cadence of 70 rpm was maintained during trials using the Computrainer® ergometer, which was calibrated as per manufacturer's instructions. Prior to all training and trial sessions participants completed a daily analysis of life demands (DALDA) questionnaire to assess stress sources (part 1) and stress symptoms (part 2) [31].

Trials, conducted at St James Highway Clinic, commenced between 1 pm and 6 pm, at least 4 h following their last meal. Heart rate was measured using a Timex® (Middlebury, USA) telemetry strap. Temperature and humidity, measured with a calibrated thermo-hygrometer (TFA-Dostmann, Mannheim, Germany), were maintained close to 20°C and 60% RH, respectively. Rating of perceived exertion was reported after 15 min into the trial and thereafter every 30 minutes. Only water was permitted during the trial. One athlete dropped out following the second trial, and did not complete the control trial. The curcumin supplement (“Meriva®” Curcumin) and corresponding identical placebo, together with respective certificate of analysis (CoA) were donated by Indena Spa. (Milan, Italy). Meriva® curcumin was chosen because of its superior bioavailability to other curcumin products. Researchers concluded that a single dosage of 376 mg of Meriva® curcumin was eighteen times superior to a standard curcumin dose of 2 g, giving a maximum plasma concentration of 207 ng/ml four hours following supplement ingestion [32]. Dosage for the present study subjects was a single dose of 500 mg of Meriva® curcumin (5 tablets) with midday meal for three days, and then 500 mg ingested just before exercise. Samples for plasma curcumin analysis were taken at the final blood sampling time only in this study, three hours post ingestion to coincide with assessment of post-exercise interleukin response.

Plasma and serum samples obtained after centrifuging were frozen at −80°C. Plasma samples for curcumin analysis were incubated for 4 hours with helix pomatia glucuronidase (Sigma Aldrich®, Delaware, USA) in a pH 5 sodium acetate buffer. This was followed by extraction with chloroform. The organic chloroform was dried under a nitrogen stream, and reconstituted in 4 ml curcumin spiked acetonitrile. These samples were then analysed for curcumin using a Waters HPLC (Milford, USA) using a method reported in literature [33]. The method was validated for identification and linearity using curcumin standard (Sigma Aldrich, Delaware, USA). Interleukins 6, 1RA, and 10 were assayed on all serum samples using ELISA kits supplied by R&D Systems Ltd (Minneapolis, USA). Haematocrit, haemoglobin concentration, white blood cell (WBC count), neutrophil proportion, cortisol concentration, and c-reactive protein concentration were all measured on blood taken immediately after exercise only (analyses were conducted by MLS laboratories, St James Hospital, Malta).

Repeated measures ANOVA was conducted with the values obtained for time, trial, and time x trial interactions. Any outliers in datasets were dealt with using Grubbs method [34]. Any significant within subject effects were then examined with the median test when data was not normally distributed (IL-6 conc.), otherwise student *t*-test was used. Ratings from the DALDA questionnaire were analysed with the wilcoxon test for paired non parametric data. Parametric results are tabulated as mean ± standard deviation, 95% confidence intervals are also given in brackets for Tables 1 and 2. Further results are graphically plotted as mean ± standard error of the mean. Median and inter-quartile range are reported when continuous data is not normally distributed. Spearman’s correlation coefficient was calculated where associations were expected. The intra-assay coefficient of variation was 9.4% for IL-6; 6.4% for IL-1RA and 3.4% for the HPLC assay of curcumin.

**Table 1:** **Ambient conditions, ergometer calibration setting and initial body mass on the day of each trial**

	**Curcumin**	**Placebo**	**Control**
**Mean ambient humidity (% RH)**	63 ± 6 (59–67)	62 ± 7 (58–66)	62 ± 6 (58–66)
**Mean ambient temperature (°C)**	19.9 ± 0.6 (19.5-20.3)	20.0 ± 0.4 (19.8-20.2)	20.1 ± 0.5 (19.8-20.4)
**Calibration value of computrainer**	2.7 ± 0.1 (2.6-2.8)	2.7 ± 0.1 (2.6-2.8)	2.8 ± 0.1 (2.7-2.9)
**Body mass (kg)**	86.7 ± 10.5 (80.5-92.9)	86.6 ± 10.4 (80.4-92.8)	87.5 ± 11.0 (80.7-94.3)
**Training (Hours×Intensity)**	11 ± 10 (5–17)	13 ± 9 (8–18)	13 ± 6 (9–17)

Habitual training load during the previous week was assessed using duration and intensity information. Training is reported in hours multiplied by intensity. Intensity was classified as low (1) medium (2) & high (3) Data are mean (± SD). Standard deviation and 95% confidence intervals are also reported following each value. No differences were noted between trials groups. Calibration value of the Computrainer is the value given to the ergometer as instructed by the manufacturer.

**Table 2:** **Physiological parameters means (± SD) measured during trial, grouped by trial type**

	**Curcumin**	**Placebo**	**Control**
**Cortisol (nMol)**	308 ± 200 (190–426)	266 ± 200 (148–384)	289 ± 228 (148–430)
**C-Reactive protein (mg/l)**	0.5 ± 0.3 (0.3-0.7)	0.9 ± 0.9 (0.4-1.4)	0.7 ± 0.6 (0.3-1.1)
**Haematocrit (%)**	43 ± 2 (42–44)	43 ± 3 (41–45)	43 ± 2 (42–43)
**Haemoglobin (g/dl)**	15.0 ± 0.7 (14.6-15.4)	14.0 ± 0.9 (13.5-14.5)	15.1 ± 0.8 (14.6-15.6)
**WBC (10** ^**9**^ **/L)**	10.1 ± 2.7 (8.5-11.7)	9.6 ± 2.5 (8.1-11.1)	10.4 ± 2.6 (8.8-12.0)
**Neutrophil (%)**	61.9 ± 9.8 (56.1-67.7)	61.4 ± 9.2 (56.0-66.8)	63.5 ± 9.5 (57.6-69.4)

Confidence intervals 95% are also reported following each value.

Parameters show no significant difference between trials. These parameters were measured only at the end of exercise. Cortisol and C - reactive protein were measured to investigate any possible effects from the active compound curcumin. Haematocrit & Haemoglobin were measured to ensure that the athletes were in similar hydration status, while white blood cell and neutrophil percentage were needed to confirm that the athlete was not suffering from an infection at the time of the trial.

## Results

All participants undertook the trial at 95% lactate threshold. Relative to W_max_ the mean power output sustained during the 2 hour ride was 54 ± 7% of the mean maximum workload (range 39% to 63% of W_max_). The humidity, temperature, and ergometer calibration values were all similar between trials (Table 1). Initial body mass and training volume were also not different between each trial (Table 1). None of the participants reported any adverse effect to supplementation or placebo ingestion. All participants reported adhering to their pre-trial diets on all trials. Participants completed all the trials in three weeks. Five participants started the trials with placebo and six with curcumin supplementation. HPLC analysis confirmed the presence of curcumin in plasma of all participants when taking the curcumin supplement. No curcumin was detected in plasma samples on other trials. Mean ± SD (range) curcumin concentration obtained was 79.7 ± 26.3 ng/ml (50.7 ng/ml to 125.5 ng/ml). The reported perceived exertion increased significantly every 30 minutes during the 2 hour ride on all trials (mean (SD) RPE was: 9 ± 1; 10 ± 2; 11 ± 2 & 12 ± 2 at 15, 45, 75 and 105 minutes during exercise; p < 0.01). There were no significant differences in RPE ratings obtained during exercise between trials (11 ± 1; 11 ± 1; 11 ± 1 for curcumin, placebo and control trials, respectively). Mean (SD) heart rate during the exercise period was also not different between trials (118 ± 12; 117 ± 10; 117 ± 13 for curcumin, placebo and control trials, respectively). Whole blood analysis of the post-exercise samples revealed no differences in cortisol, c-reactive protein, haematocrit, haemoglobin, WBC, or neutrophil proportion between trials (Table 2).

**Figure 2:**
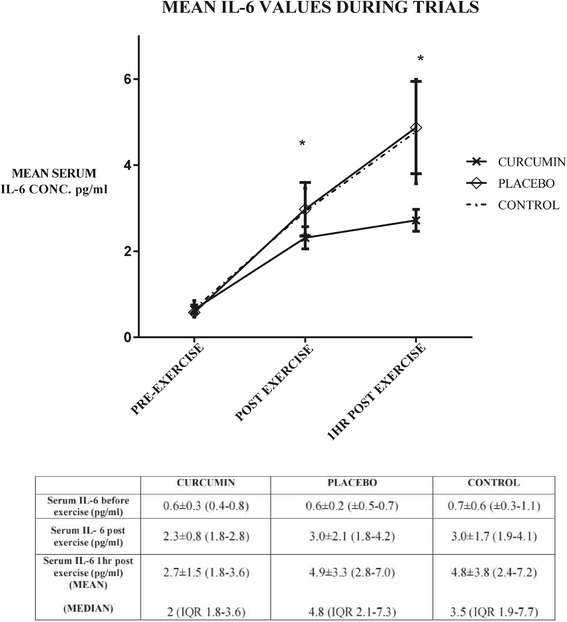
**Mean (±SEM) IL-6 concentration obtained before exercise, immediately after exercise, and one hour following exercise on each trial day.** *indicates significant difference from pre-exercise on all trials. No statistical significant difference between interleukin 6 values was observed. Table shows mean cytokine levels, standard deviation, and 95% confidence intervals during trials. Median and interquartile range IQR are also shown for 1 hour post exercise.

**Figure 3:**
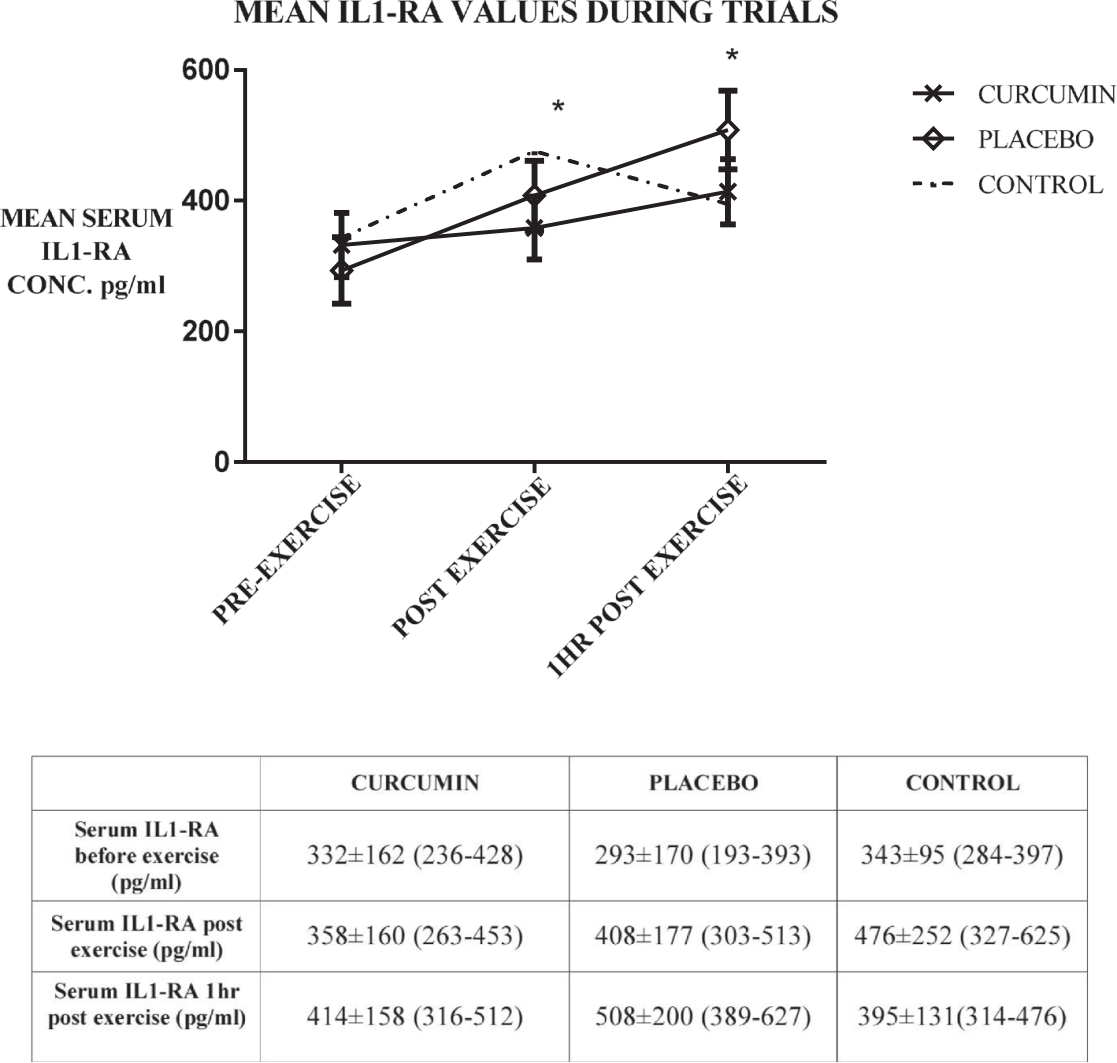
**Mean (±SEM) IL1-RA concentration obtained before exercise, immediately after exercise, and one hour following exercise on each trial day.** *indicates significant difference from pre-exercise. Table shows mean cytokine levels, standard deviation, and 95% confidence intervals during trials.

**Figure 4:**
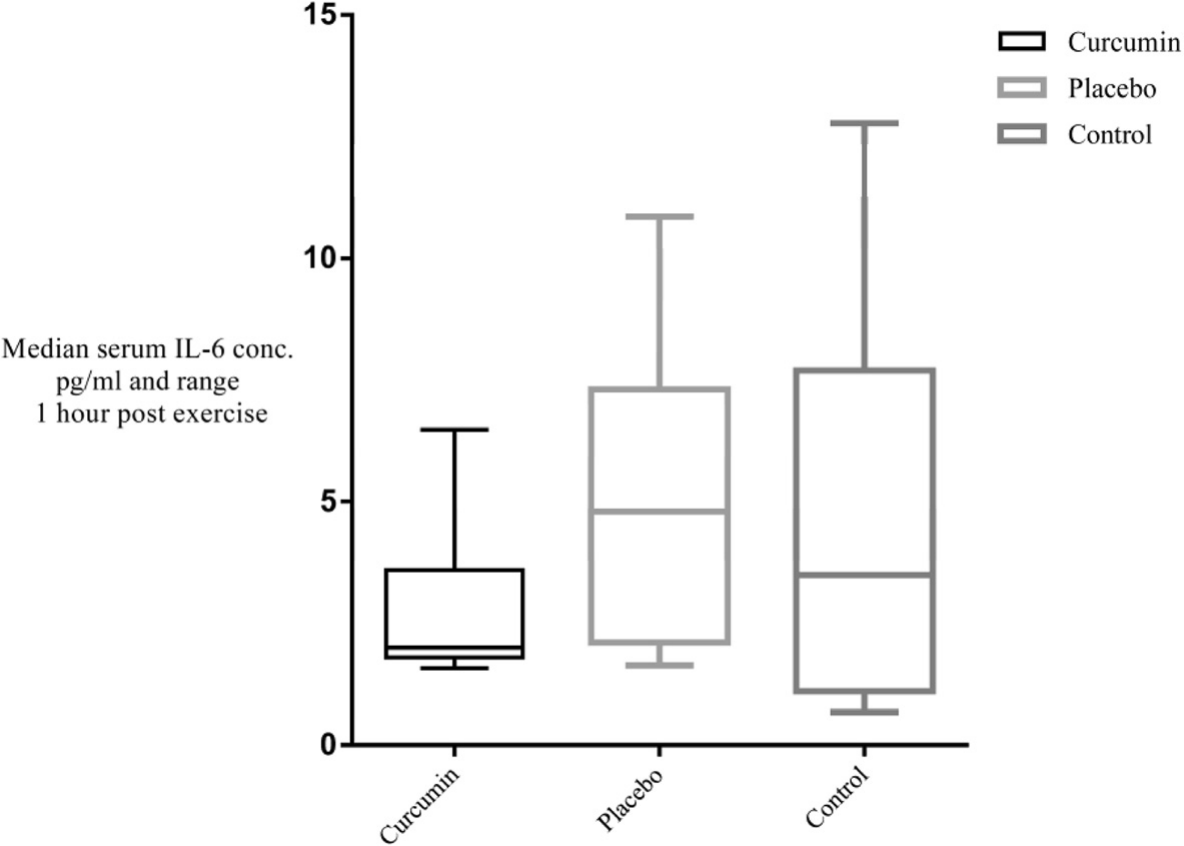
**Median IL-6 concentration and range one hour post exercise for curcumin, placebo and control trials.** Note curcumin dataset still positively skewed (towards low values) despite removing an outlier.

Serum IL-6 data demonstrated a tendency for an interaction effect (time x trial interaction p = 0.06; F = 4.03) between curcumin and placebo trials (Figure 2). Curcumin only appeared to lower the concentration of IL-6 released one hour following exercise when compared to placebo, but this failed to reach statistical significance (p = 0.18; n = 10; 95% C.I. 1.63 ≤ × ≤ 3.81) (Figure 3). Estimation of size of effect proves difficult because one set of data is not normally distributed. Nonetheless Cohen’s d is of 0.84 hinting at a possibly large effect (Figure 4). The correlation analysis revealed a significant association (p = 0.02) between IL-6 elevation and percentage W_max_ power output sustained during the exercise task (correlation coefficient rho 0.41 (dƒ = 30)). No association was observed between attenuation of IL-6 response following exercise with the plasma concentration levels of curcumin (p = 0.92; correlation coefficient rho −0.04 (dƒ = 9)). There was no difference between the trials for IL1-RA (time x trial interaction p = 0.85, (F = 0.44) when analysing the ANOVA for repeated measures. Correlation coefficient between percentage W_max_ power and change in IL1-RA concentration was 0.34 (dƒ = 30), but failed to reach statistical significance (p = 0.06). There was no detectable increase in IL-10 on any of the trials.

The DALDA questionnaire (Table 3) revealed a higher number of “better than usual” results on the training day when ingesting curcumin compared to placebo and control. This was statistically significant between placebo and supplementation in both stress sources (Part 1, p = 0.04) and stress symptoms (Part 2, p = 0.04). The number of “better than usual” results obtained between curcumin and control on the training day was also higher but not statistically significant (Part 1,p = 0.06; and Part 2, p = 0.14). There were no differences in scoring on Part 1 or Part 2 of the DALDA questionnaire between treatments on the trial days.

**Table 3:** **DALDA (Daily Analysis of Life Demands on Athletes) questionnaire responses (median & range) for both training and trial days**

	**DALDA part 1 (training day) stress sources**			**DALDA (part 2 training day) stress symptoms**		
*RESPONSE*	*A (Worse)*	*B (Same)*	*C (Better)*	*A (Worse)*	*B (Same)*	*C (Better)*
CURCUMIN (n = 11)	0 (0–3)	4 (4–9)	3 (0–5)^†^	1 (0–4)	21(4–25)	3 (0–19)†
PLACEBO (n = 11)	1 (0–5)	7 (2–9)	1 (0–6)	2 (0–7)	20 (8–25)	2 (0–15)
CONTROL (n = 10)	2 (0–3)	6 (2–9)	0 (0–7)	2 (0–9)	22 (5–25)	2 (0–18)
	**DALDA part 1 (trial day) stress sources**			**DALDA part 2 (trial day) stress symptoms**		
*RESPONSE*	*A (Worse)*	*B (Same)*	*C (Better)*	*A (Worse)*	*B (Same)*	*C (Better)*
CURCUMIN (n = 11)	2 (0–4)	6 (2–9)	1 (0–7)	2 (0–5)	22 (5–25)	1 (0–20)
PLACEBO (n = 11)	1 (0–5)	8 (2–9)	0 (0–6)	1 (0–6)	23 (5–25)	0 (0–18)
CONTROL (n = 10)	2 (0–3)	7 (3–9)	0 (0–6)	3 (0–8)	22 (4–25)	1 (0–19)

Data is grouped according to trial type. A – Worse than usual; B – Same as usual; C – Better than usual. ^†^indicates statistical significant difference between curcumin and placebo trials, p-value in both parts between curcumin and placebo is 0.04 using Wilcoxon signed ranks.

## Discussion

The current study has not revealed a statistically significant difference between the supplementation with curcumin vs. placebo or control. However these results suggest that a positive inhibitory effect of curcumin on IL-6 production/release or an enhanced uptake *in vivo could occur a*t higher supplementation doses, and under different trial conditions (suggested underneath). These observations, although again not statistically significant, lend some support to the previous cell and animal model data, and suggest that further studies in humans may be warranted. The lack of statistical significance in our dataset suggests that sample size, mode of exercise, intensity of exercise, dose of curcumin, or sample collection times are interesting issues for discussion and further investigation.

Sample size estimates using the mean difference 1 hr post-exercise, and standard deviation, from the present study indicate that adequate power could be obtained with 26 participants. Given the large variance in response of IL-6 post-exercise within the current study it would be of interest to analyse responses in a similar trial on a group who may provide a more homogeneous response. The recruitment of cyclists also may have limited our ability to observe any possible effect of curcumin on post-exercise cytokine concentration, due to the absence of eccentric contractions or weight bearing impact during the exercise task. A two hour long exercise regimen was chosen because duration of exercise is considered a better predictor of serum interleukin elevation than intensity. Running is associated with a higher rise in cytokine concentration post-exercise than observed following cycling [30], and may therefore be a mode of choice in future studies.

Despite the light exercise intensity examined in the present study a response in IL-6 and IL1-RA was still elicited, primarily because the exercise was of sufficient duration. We deliberately adopted a low carbohydrate diet in an attempt to exacerbate the cytokine response to prolonged cycling exercise [12], and this seems to have been effective. Participants whose trial was at a higher workload intensity relative to their maximum workload capacity had a greater increase in IL1-RA and IL-6 concentration one hour after exercise. This was statistically significant for IL-6, and close to statistical significance for IL1-RA. It is important to note that some studies observing higher cytokine responses employed a performance time/distance trial following a period of cycling at a submaximal steady state intensity [12]. This type of protocol would enhance the cytokine response post-exercise, and indicates that higher intensity bouts may be of most interest in future studies examining curcumin effects on cytokine response.

Although our data indicate no statistically significant effect of curcumin supplementation on IL-6 and IL1-RA response to exercise, this could be due to the curcumin dose and plasma curcumin response. Cuomo and colleagues previously indicated that serum micro-molar concentrations of curcumin would likely be necessary for pharmacological *in vivo* effects [32]. Indeed, it is possible that a significant effect on post-exercise interleukin concentrations would have been achieved with a higher plasma curcumin concentration in the present study. The curcumin concentration achieved in the present study was almost 80 ng/ml (0.22 μmoles/L). A recent paper observed an effect of curcumin on plasma oxidative stress markers following exertion in humans when plasma curcumin concentration was elevated to around 100 ng/ml [35]. Recent work [36] has demonstrated that intraperitoneal injection of curcumin counteracts muscle atrophy in rats possibly also through anti-oxidant actions. It is unclear if such effects can be demonstrated in a human model and what dose of curcumin would be required to achieve this, but translation to a human model could provide relevant outcomes for sport or clinical practice. It is, therefore, recommended that future studies quantify the plasma concentration of curcumin required to achieve significant clinically relevant outcomes and investigate any possible association with anti-oxidant activity. Furthermore, blood sampling after 2, 16, & 24 hours following exercise would have provided further data on cortisol, C-reactive protein, and interleukin 10 responses which are known to be influenced by circulating IL-6 concentration, but at a later time than the last blood sample taken in our study.

Ratings of perceived exertion significantly increased throughout the exercise period on all trials. Given the prior glycogen depleting exercise during the training day, and prescribed low carbohydrate diet, it is likely that glycogen depletion contributed towards increased ratings of exertion during the exercise period. The DALDA results indicate that participants felt better on the second day of curcumin supplementation (the training day). The number of “better than usual” responses was higher than placebo and control on the second day of supplementation. It is important to note that DALDA is a retrospective tool of psychological causes and symptoms [31], while that ratings of perceived exertion (RPE) is a prospective tool assessing the extent of exercise difficulty. The study was aimed to provide the same repeatable exercise stressor every time the trial was repeated conducted with curcumin, placebo, and control. The fact that the RPE has did not changed to the extent of its sensitivity, between experimental variables provides evidence that the study managed to reproduce similar exercise conditions in all trials. A study on patients suffering from osteoarthritis taking 1 g of curcumin supplementation for eight months showed less pain and better movement reported by patients taking the supplementation versus placebo [37]. Moreover our views are supported by a recent study on curcumin supplementation and delayed muscle onset soreness (DOMS). This study has demonstrated that, participants taking 400 mg curcumin supplementation for 2 days, report less DOMS than participants taking placebo [38]. The authors suggest that potentially acute curcumin supplementation may be of use to help participants with higher intensity training workloads.

Interestingly, researchers have recently described significant anti-inflammatory effects of curcumin, and have confirmed that curcumin acts to inhibit lipopolysaccharide stimulated NF-κB, reduce IL-6, and reduce PMA induced reactive oxygen species (ROS) production, in human neutrophils [39]. Furthermore, others [40] have noted a significant attenuation in skeletal muscle IL-6 mRNA during exercise with anti-oxidant vitamin supplementation (vitamin C and E); and lead to significantly decreased plasma IL-6 concentration. Starkie and colleagues note a significant reduction in plasma IL-6 but not in skeletal muscle IL-6 mRNA following carbohydrate intake [41]. These observations suggest that measurement of early events in cytokine production are important to monitor in future human studies, and that concurrent supplementation of carbohydrate alongside an anti-oxidant like curcumin might have a superior effect to that of carbohydrate on its own on attenuation of cytokine response following exercise. It must be noted that subjects in our study were glycogen depleted, and that further studies are needed to confirm or refute similar findings or trends in athletes who are carbohydrate replete. As such the usefulness of curcumin supplementation during competition or training needs to be studied separately.

The present study also included a control arm, intended to identify any placebo effects, especially in subjective measures and to help confirm any trends observed with curcumin supplementation. No statistical difference between placebo and control values was found for any variables and no difference was observed from control in those who commenced the study with either curcumin supplementation, or placebo supplementation. This suggests both that the washout period was sufficiently long between trials with our present protocol and that trial stress was adequately reproduced.

## Conclusion

There is considerable debate concerning the impact of blunting cytokine and inflammatory marker responses to exercise on the adaptive stimulus to exercise [42], and further work is required to determine the effects of blunting cytokine and inflammatory marker responses to exercise on incidence of infection, and training adaptation in athletes. Given that the limited bioavailability of the polyphenol curcumin has been now improved with new preparations as used in the present study, it would seem prudent to direct more research towards athletic and clinical populations. In conclusion, the results from the present study did not reveal any statistical difference between intervention and placebo. However our interpretation based on the findings presented in this paper does not exclude the possibility of an attenuating effect on IL-6 by curcumin. This is supported by the results obtained in this study and corroborated by findings in other published studies. We conclude that the effect of curcumin supplementation on interleukins and other inflammatory markers needs to be further investigated with observations in a larger sample including examination of exercise mode, intensity effects, and curcumin dose effects.
